# *Parachlamydia* spp. and Related *Chlamydia*-like Organisms and Bovine Abortion

**DOI:** 10.3201/eid1312.070655

**Published:** 2007-12

**Authors:** Nicole Borel, Silke Ruhl, Nicola Casson, Carmen Kaiser, Andreas Pospischil, Gilbert Greub

**Affiliations:** * University of Zurich, Zurich, Switzerland; † University of Lausanne, Lausanne, Switzerland

**Keywords:** Obligate intracellular bacteria, chlamydia, chlamydia-like organisms, chlamydia-related bacteria, Chlamydiales, pathogenicity, dispatch

## Abstract

*Chlamydophila abortus* and *Waddlia chondrophila* cause abortion in ruminants. We investigated the role of *Parachlamydia acanthamoebae* in bovine abortion. Results of immunohistochemical analyses were positive in 30 (70%) of 43 placentas from which *Chlamydia*-like DNA was amplified, which supports the role of *Parachlamydia* spp. in bovine abortion.

Chlamydiae are implicated in a wide variety of clinically and economically important diseases in livestock and companion animals. *Chlamydophila pecorum* has been associated with abortion, conjunctivitis, encephalomyelitis, enteritis, pneumonia, and polyarthritis in ruminants, and *Cp*. *abortus* infection is the most common cause of abortion in sheep and goats (*1*). *Cp*. *abortus* also causes zoonotic infection in humans, which in pregnant women, can result in spontaneous abortion (*2*,*3*).

During the past decade, new *Chlamydia*-like organisms have been discovered and now emerge as possible public health threats. *Simkania negevensis* is considered a possible emerging agent of pneumonia (*4*), and evidence supports the role of *Parachlamydia acanthamoebae* as an agent of pneumonia (*5*,*6*). *Waddlia chondrophila* is another *Chlamydia*-like organism initially isolated from lung, liver, and other tissues of an aborted bovine fetus in the United States (*7*). This organism is now considered an abortigenic agent with a worldwide distribution in cattle, as shown by a recent report of *Waddlia*-related abortion in Germany (*8*).

The role of *Chlamydia*-like organisms in bovine abortion is further supported by results of a study of abortion in cattle in Graubünden, Switzerland (*9*). Analysis of placental specimens by PCR showed that 43 (18.3%) of 235 placentas contained DNA from *Chlamydia-*like organisms (*9*). Of these 43 specimens, 8 showed sequence similarity to *P*. *acanthamoebae* (95%–99%). Identification was not possible in the remaining 35 specimens because of their strong sequence similarity with uncultured chlamydial DNA sequences (Table). These 35 specimens were referred to as *Chlamydia*-like organisms. None of these 35 specimens was positive by immunohistochemical analysis with antibodies against *Chlamydiaceae*. This finding indicates that routine diagnostic approaches based on chlamydial lipopolysaccharide would not detect most *Chlamydia*-like infections (*9*). To confirm the role of these novel chlamydiae in bovine abortion, we analyzed these placental samples from cattle in Switzerland by using a new specific immunohistochemical protocol and transmission electron microscopy.

**Table:** Results of histologic, 16S rRNA sequence, and immunohistochemical analyses for 43 placentas positive for *Chlamydia*-like DNA by a 16S rRNA PCR*

Specimen no.	Histology			16S rRNA sequence†			Immunohistochemistry	
	Placentitis	Vasculitis		Species	% Similarity		*Parachlamydia* spp.	*Waddlia*
1	N	Yes		*Parachlamydia*	99		+	–
2	N	No		*Parachlamydia*	97		+	–
3	P/N	No		*Parachlamydia*	98		+	–
4	P/N	No		*Parachlamydia*	97		–	–
5	P/N	No		*Parachlamydia*	97		–	–
6	A	No		*Parachlamydia*	96		+	–
7	A	No		*Parachlamydia*	96		+	–
8	A	No		*Parachlamydia*	97		+	–
9	P/N	Yes		*Chlamydia*-like	92		–	–
10	P/N	Yes		*Chlamydia*-like	92		–	–
11	P/N	Yes		*Chlamydia*-like	93		–	–
12	P/N	Yes		*Chlamydia*-like	91		+	–
13	P/N	No		*Chlamydia*-like	82		+	–
14	P/N	No		*Chlamydia*-like	91		+	–
15	P/N	No		*Chlamydia*-like	92		+	–
16	P/N	No		*Chlamydia*-like	92		+	–
17	P/N	No		*Chlamydia*-like	92		+	–
18	P/N	No		*Chlamydia*-like	92		+	–
19	P/N	No		*Chlamydia*-like	92		+	–
20	P/N	No		*Chlamydia*-like	92		+	–
21	P/N	No		*Chlamydia*-like	93		+	–
22	P/N	No		*Chlamydia*-like	94		+	–
23	P/N	No		*Chlamydia*-like	95		+	–
24	P/N	No		*Chlamydia*-like	100		+	–
25	P/N	No		*Chlamydia*-like	93		–	–
26	P/N	No		*Chlamydia*-like	93		–	–
27	P/N	No		*Chlamydia*-like	95		–	–
28	P/N	No		*Chlamydia*-like	96		–	–
29	N	No		*Chlamydia*-like	85		+	–
30	N	No		*Chlamydia*-like	88		+	–
31	N	No		*Chlamydia*-like	88		+	–
32	N	No		*Chlamydia*-like	91		+	–
33	N	No		*Chlamydia*-like	91		+	–
34	N	No		*Chlamydia*-like	95		+	–
35	P	No		*Chlamydia*-like	91		+	–
36	P	No		*Chlamydia*-like	94		+	–
37	A	No		*Chlamydia*-like	91		+	–
38	A	No		*Chlamydia*-like	92		+	–
39	A	No		*Chlamydia*-like	92		+	–
40	A	No		*Chlamydia*-like	91		–	–
41	A	No		*Chlamydia*-like	92		–	–
42	A	No		*Chlamydia*-like	93		–	–
43	A	No		*Chlamydia*-like	95		–	–

*When partial 16S rRNA sequence showed a similarity >95% with a recognized species (i.e., *Parachlamydia acanthamoebae*), the corresponding genus was reported (i.e., *Parachlamydia* spp.). Conversely, when the sequence showed a best BLAST (www.ncbi.nlm.nih.gov) hit with uncultured or uncharacterized *Chlamydia*-related organisms, the sequence was designated as being similar to a *Chlamydia-*like organism. N, necrotizing; +, positive; –, negative; P, purulent; A, autolysis. †A 278-bp fragment was amplified and sequenced (*9*).

## The Study

Formalin-fixed and paraffin-embedded placenta specimens were analyzed by using histopathologic and immunohistochemical techniques. Hematoxylin and eosin–stained histologic sections of all placenta specimens (n = 235) were examined for the type and degree of placentitis or vasculitis. Paraffin-embedded sections of specimens positive for *Chlamydia*-like organisms by 16S rRNA PCR (n = 43) were analyzed for *Parachlamydia* spp. and *Waddlia* by using specific mouse polyclonal antibodies as described (*10*). Optimization experiments for immunohistochemical analysis were performed by using infected amebal and infected HEp-2 cell pellets. Briefly, *Acanthamoeba castellanii* cultures were infected with *P*. *acanthamoebae* strain Hall coccus and *W*. *chondrophila* strain ATCC 1470. HEp-2 cell monolayers were infected with *Cp*. *abortus* strain S26/3. Uninfected cells were used as negative controls. Amebal and cell pellets were prepared as described (*11*). Optimization of the immunohistochemical protocol for experimentally infected amebal pellets showed the species specificity of mouse antibodies to *P*. *acanthamoebae* and *W*. *chondrophila*. We did not observe cross-reactivity of both antibodies with *Cp*. *abortus*–infected HEp-2 cell pellet (data not shown).

To test placental specimens, we used mouse polyclonal antibody against *P*. *acanthamoebae* and *W*. *chondrophila* at dilutions of 1:1,000 and 1:2,000, respectively. Antigen detection was performed with the ChemMate Detection Kit (Dako, Glostrup, Denmark) according to the manufacturer’s instructions. Briefly, paraffin-embedded sections were deparaffinated in xylene and rehydrated through graded ethanol to water. Antigen was detected by using repeated microwave heating (750 W for 10 min) in citrate buffer, pH 6.0 (Target Retrieval Solution, Dako). Specimens (slides) and primary antibodies were incubated for 1 hour. Negative and positive controls of each section were included as described (*9*).

Histopathologic lesions such as purulent or necrotizing placentitis were observed in 149 (63.4%) of 235 specimens. Placentitis was observed in 5 of 8 specimens positive for *P*. *acanthamoebae*, and vasculitis was observed in 1 of 8 specimens (Table). Positive antigen labeling was observed in 6 of 8 specimens for *Parachlamydia* spp., but antigen labeling was negative in all specimens for *Waddlia* (Table). The Figure, panel A shows positive immunohistochemical labeling in 1 of these specimens. Among the 35 placentas positive by PCR for *Chlamydia*-like organisms other than *P*. *acanthamoebae*, 28 (82.3%) showed obvious purulent or necrotizing placentitis by histologic analysis. Four of the 28 specimens with placentitis also had vasculitis. A total of 24 (68.6%) of 35 specimens were positive when tested with antibody against *P*. *acanthamoebae*, and all 35 specimens were negative when tested with antibody against *W*. *chondrophila*.

**Figure:**
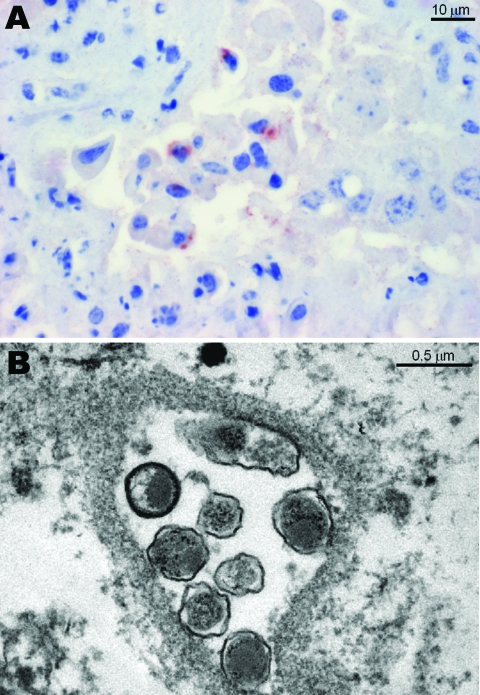
A) Immunohistochemical analysis of a bovine placenta positive by PCR for *Parachlamydia acanthamoebae*, showing a positive brown-red granular reaction within trophoblastic epithelium. Antigen detection was conducted with a polyclonal antibody against *Parachlamydia* spp. (3-amino-9-ethylcarbazole/peroxidase method, hematoxylin counterstain). B) Transmission electron micrograph of bovine placenta positive by PCR and immunohistochemical analysis for *P*. *acanthamoebae*, showing 7 cocci-shaped bacteria in an inclusion with morphologic features similar to those of *Chlamydia*-like organisms (*12*).

Two placental specimens positive for *Parachlamydia* spp. by immunohistochemical analysis and 16S rRNA PCR were further investigated by transmission electron microscopy for ultrastructural evidence of *Chlamydia*-like organisms. Briefly, placental tissue specimens were fixed with glutaraldehyde and osmium tetroxide and embedded in Epon resin. Ultrathin sections (80 nm) were mounted on gold grids (Merck Eurolab, Dietlikon, Switzerland), contrasted with uranyl acetate dihydrate (Fluka, Buchs, Switzerland) and lead citrate (lead nitrate and tri-natrium dehydrate, Merck Eurolab), and analyzed with a Philips (Eindhoven, the Netherlands) CM10 electron microscope. Both placentas showed *Chlamydia*-like structures (Figure, panel B).

## Conclusions

To our knowledge, this is the first description of *Parachlamydia* spp. in bovine abortion. The organism was detected by PCR (*9*) and within placental lesions by immunohistochemical analysis by using an antibody specific for *Parachlamydia* spp. and electron microscopy. All specimens were negative for *Waddlia* by immunohistochemical analysis. Isolation of *Parachlamydia* spp. from aborted bovines is necessary to confirm that this agent causes bovine abortion. *Parachlamydia* spp. may be involved in lower respiratory tract infections in humans (*5*,*6*) and may replicate within both pneumocytes (*13*) and human macrophages (*14*). Thus, caution should be taken when handling bovine abortion material because of the potential zoonotic risk.
